# Neurosarcoidosis presenting with panhypopituitarism: diagnostic and therapeutic challenges: a case report

**DOI:** 10.1186/s13256-026-06196-4

**Published:** 2026-06-12

**Authors:** Moazama Shakeel Ahmed, Talha Kashif, Anurag Jha, Muzamil Khan, Hammad Javaid, Hafiz Fahad Ullah Saeed, Hasibullah Aminpoor

**Affiliations:** 1 Icahn School of Medicine at Mount Sinai/Queens, Queens, NY USA; 2 King Edward Medical University, Lahore, Pakistan; 3 The George Washington University School of Medicine and Health Sciences, Washington, DC USA; 4 Faculty of Medicine, Kabul University of Medical Sciences “Abu Ali Ibn Sina”, Kabul, Afghanistan; 5 Department of Internal Medicine, Rabia Balkhi National Complex Hospital, Kabul, Afghanistan

**Keywords:** Neurosarcoidosis, Panhypopituitarism, Hypothalamic–pituitary axis, Sarcoidosis, Diabetes insipidus

## Abstract

**Background:**

Neurosarcoidosis is a rare but serious manifestation of sarcoidosis involving the central nervous system. When it affects the hypothalamic–pituitary axis, it can result in panhypopituitarism and diabetes insipidus due to disruption of anterior and posterior pituitary hormone regulation. Early recognition is critical, as delayed diagnosis may lead to life-threatening complications.

**Case presentation:**

We report the case of a 42-year-old White man with a 12-year history of sarcoidosis who presented with abdominal pain, hypotension, hypothermia, hypernatremia, polyuria, and polydipsia. He also had widespread skin plaques, joint pain, and cartilage deformities. Notably, he had developed drug-resistant seizures 3 years earlier. Laboratory investigations demonstrated hypernatremia, low urine osmolality, and hormonal deficiencies consistent with panhypopituitarism. Imaging revealed suprasellar and brainstem lesions. After excluding alternative diagnoses, neurosarcoidosis was established. The patient responded well to hormone replacement therapy with levothyroxine and desmopressin, which led to clinical improvement.

**Conclusion:**

This case highlights the importance of considering neurosarcoidosis in patients with sarcoidosis who develop endocrine or neurological symptoms. It underscores the value of timely diagnosis and multidisciplinary management to prevent irreversible complications and improve long-term outcomes.

## Introduction

Sarcoidosis is a chronic granulomatous disease of unknown etiology characterized by the formation of granulomas without central necrosis [1]. It usually occurs as a consequence of chronic immune response, possibly triggered by environmental factor in genetically susceptible individuals resulting in the activation of CD4+ lymphocytes and macrophages and formation of non-caseating granulomas [2]. It can affect almost any part of the body with lung, skin, and eyes being commonly involved [1].

Central nervous system involvement results in a rare but serious manifestation of sarcoidosis also known as the neurosarcoidosis. It occurs in approximately 5–15% of sarcoidosis patients [3]. It can lead to wide range of complications like cranial nerve palsies, cognitive impairment, seizures, and hydrocephalus, mimicking other diseases and making its diagnosis and treatment complicated [4].

Neurosarcoidosis can affect the hypothalamus and pituitary gland; hypothalamic–pituitary involvement is estimated to occur in approximately 0.5% of all sarcoidosis patients, though pituitary dysfunction has been reported in 12–17% of patients with established neurosarcoidosis, highlighting its clinical significance within this subgroup. This results in the infiltration of non-caseating granulomas and disruption of the hypothalamic–pituitary axis [3, 5]. This can result in the spectrum of endocrine deficiencies including diabetes insipidus, anterior pituitary hormone deficiencies, and in severe cases panhypopituitarism which is the deficiency of all hormones of the pituitary gland [5].

Here, we present a rare case of neurosarcoidosis with panhypopituitarism in 42-year-old male patient with history of sarcoidosis focusing on clinical presentation along with diagnostic and treatment approaches. Although neurosarcoidosis-associated panhypopituitarism has been described in recent literature, this case presents a distinctive clinical phenotype characterized by delayed neuroendocrine presentation 12 years after initial diagnosis, concurrent drug-resistant seizures, and prominent autonomic features, a combination not well represented in prior reports. This case further illustrates the practical application of current diagnostic criteria and the management of concurrent adrenal insufficiency and diabetes insipidus in a multidisciplinary setting. This case report has been reported following the CARE criteria for medical case reports [6].

## Case presentation

A 42-year-old White male with a history of sarcoidosis, diagnosed in 2013 via skin biopsy, presented with abdominal pain, hypernatremia, hypothermia, and hypotension. He also reported polyuria and polydipsia, symptoms that had been present for approximately 6 months prior to this admission, suggesting a subacute onset of central diabetes insipidus. His medical history was notable for chronic prednisone 10 mg daily and methotrexate 15 mg weekly use for sarcoidosis, T3–T6 vertebral compression fractures secondary to long-term steroid therapy, and refractory seizures for the past 3 years despite valproic acid treatment.

On examination, he had widespread raised reddish plaques, joint pain with knee effusion, and cartilage deformities involving the nasal, palatal, and laryngeal structures.

### Diagnostic assessment

Laboratory testing (Table 1) revealed elevated serum sodium (148 mEq/L [148 mmol/L]), high serum osmolality (305 mOsm/kg H₂O), low urine osmolality (94 mOsm/kg H₂O), and low urine specific gravity (1.005), consistent with diabetes insipidus. Endocrine evaluation showed low 8 AM cortisol (0.9 μg/dL), suppressed TSH (0.03 μIU/mL) with low-normal free T4 (0.7 ng/dL), low LH (< 0.1 mIU/mL), low free testosterone (< 3 ng/dL), and mildly elevated prolactin (20.3 ng/mL), confirming panhypopituitarism.

**Table 1:** Significant Lab Results

Parameter	Value (SI unit)	Normal range (SI unit)
Serum Na+	148 mEq/L (1.48 × 10^8^ nmol/L)	136–146 mEq/L (1.36 × 10^8^ nmol/L–1.46 × 10^8^ nmol/L)
Serum osmolality	305 mOsm/kg (3.05 × 10^8^ nmol/kg)	275–295 mOsm/kg (2.75 × 10^8^ nmol/kg–2.95 × 10^8^ nmol/kg)
Urine osmolality	94 mOsm/kg (9.4 × 10^7^ nmol/kg)	50–1200 mOsm/kg (5 × 10^7^ nmol/kg–1.2 × 10^9^ nmol/kg)
Urine specific gravity	1.005	1.005–1.030
8 am serum cortisol	0.9 µg/dL (24.83 nmol/L)	6.0–18.4 µg/dL (165.54–507.64 nmol/L)
Serum free testosterone	< 3 ng/dL (< 0.104 nmol/L)	300–1080 ng/dL (10.40–37.45 nmol/L)
Luteinizing hormone	< 0.1 mIU/mL	0.6–12 mIU/mL
Follicle-stimulating hormone	3.1 mIU/mL	1.6–9.74 mIU/mL
Free T4	0.7 ng/dL (0.009 nmol/L)	0.61–1.12 ng/dL (0.008–0.014 nmol/L)
Thyroid-stimulating hormone	0.03 µIU/mL	0.34–5.60 µIU/mL
Serum prolactin	20.3 ng/dL (0.0088 nmol/L)	3.5–19 ng/dL (0.0015–0.0083 nmol/L)

Na⁺: Sodium; mEq/L: Milliequivalents per liter; mOsm/kg: Milliosmoles per kilogram; µg/dL: Micrograms per deciliter; ng/dL: Nanograms per deciliter; mIU/mL: Milli-international units per milliliter; ng/mL: Nanograms per milliliter; µIU/mL: Micro-international units per milliliter; nmol/L and nmol/kg: Nanomoles per liter or kilogram, respectively, calculated from conventional units using molecular weights (for hormones) or direct molar equivalence (for electrolytes and osmolality)

**Fig. 1:**
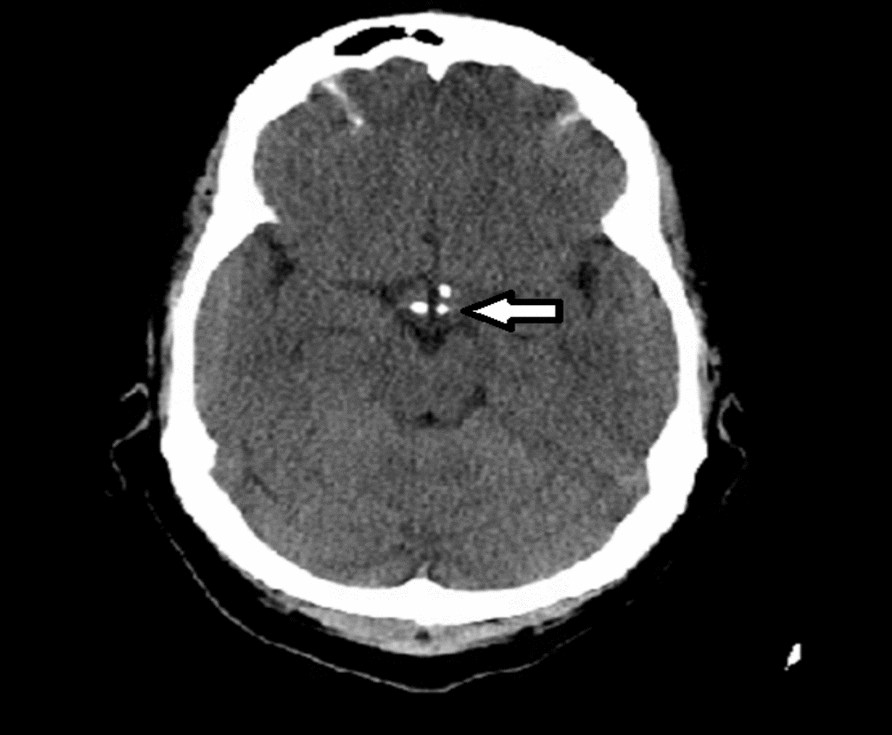
Axial Computed Tomography (CT) image of the brain showing dot-like hyperdense foci with calcifications in the suprasellar cisternal region, suggestive of neurosarcoid granulomatous infiltration

**Fig. 2:**
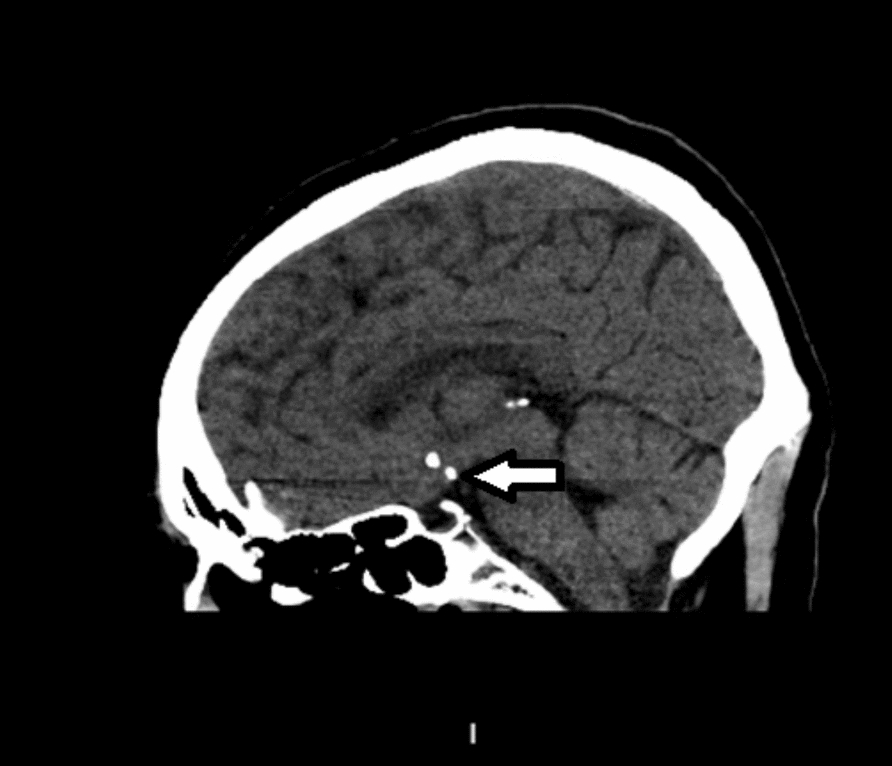
Sagittal Computed Tomography (CT) image of the brain highlighting involvement of the hypothalamic-suprasellar area with abnormal attenuation, consistent with granulomatous disease

**Fig. 3:**
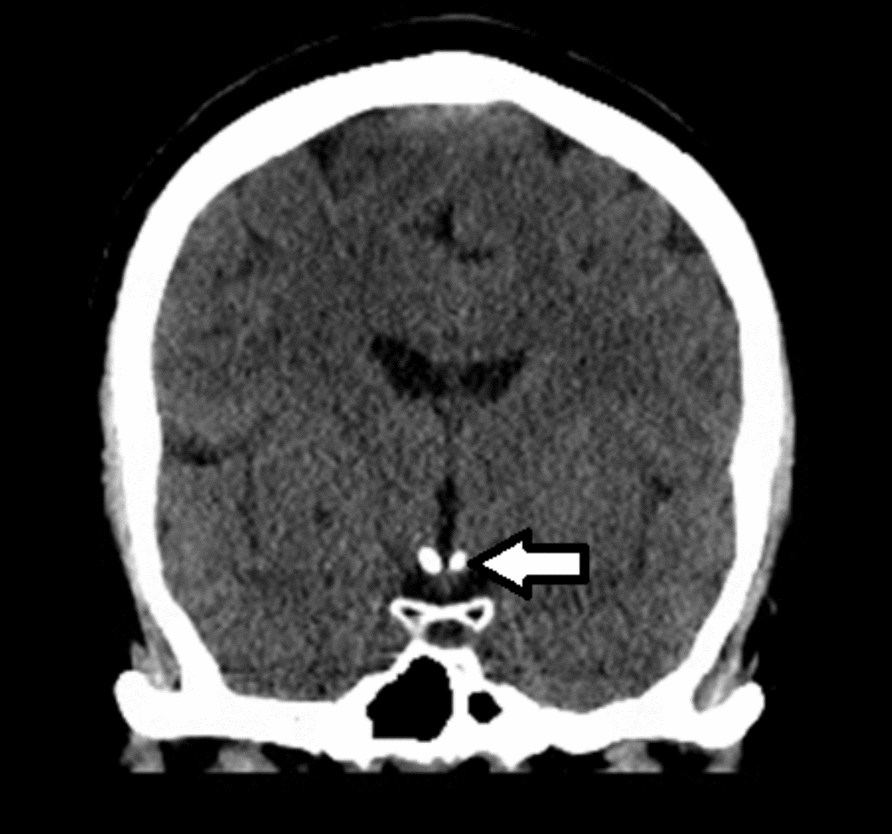
Coronal Computed Tomography (CT) image of the brain demonstrating symmetrical dot-like enhancements in the suprasellar region, characteristic of neurosarcoidosis affecting the pituitary axis

Brain CT (Figs. 1, 2, 3) demonstrated dot-like enhancements in the brainstem and suprasellar area. Review of prior MRI from another facility revealed lesions involving the optic pathway, hypothalamus, brainstem, and upper cervical cord. CSF analysis was unremarkable, and infectious (for example, tuberculosis) and neoplastic etiologies were excluded. The findings were consistent with neurosarcoidosis involving the hypothalamic–pituitary axis.

### Treatment

The patient continued prednisone 10 mg daily and methotrexate 15 mg weekly at previous doses for sarcoidosis. Given the critically low 8 AM cortisol of 0.9 μg/dL, consistent with severe secondary adrenal insufficiency, the patient was initiated on acute stress-dose hydrocortisone (100 mg intravenous bolus, followed by 50 mg every 8 h), which was subsequently transitioned to maintenance oral hydrocortisone (20 mg in the morning and 10 mg in the afternoon) prior to discharge. It is acknowledged that chronic prednisone use for systemic sarcoidosis may have contributed to HPA axis suppression in addition to direct hypothalamic–pituitary infiltration. Endocrinology initiated levothyroxine 100 μg daily for central hypothyroidism. For diabetes insipidus, desmopressin 0.05 mg (50 μg) orally once daily was started, resulting in a rise in urine osmolality from 94 to 448 mOsm/kg within 24 h.

### Outcome and follow-up

The patient’s electrolyte abnormalities improved, and his symptoms stabilized during hospitalization. He was discharged with plans for outpatient endocrinology follow-up and re-evaluation of pituitary hormone levels. Long-term hormone replacement therapy was anticipated given the likelihood of irreversible pituitary damage in neurosarcoidosis. At 6-month outpatient follow-up, the patient remained on levothyroxine 100 μg daily, desmopressin 0.05 mg (50 μg) orally once daily, and maintenance hydrocortisone. Repeat hormonal evaluation confirmed persistent panhypopituitarism with no evidence of recovery of any pituitary axis, consistent with the known irreversibility of granulomatous pituitary damage. Seizure frequency showed partial improvement with optimized antiepileptic therapy alongside continued immunosuppression; however, complete seizure freedom was not achieved, reflecting the refractory nature of extensive CNS involvement. The immunosuppressive regimen was under active reassessment by the multidisciplinary team.

## Discussion

Neurosarcoidosis with hypothalamic–pituitary represents a rare yet severe manifestation of systemic sarcoidosis that often leads to irreversible endocrine dysfunction [7]. Our patient a 42-year-old male with long-standing systemic sarcoidosis, presented with panhypopituitarism, diabetes insipidus, and refractory seizures due to neurosarcoidosis. Imaging revealed hypothalamic–pituitary involvement and endocrine workup confirmed deficiencies in cortisol, TSH, LH, and ADH.

This case is distinguished from prior reports of hypothalamic–pituitary neurosarcoidosis in several notable respects. First, the neuroendocrine complications manifested 12 years after the initial sarcoidosis diagnosis, illustrating that delayed presentation is possible even in patients previously considered stable on immunosuppressive therapy. Second, the concurrent presence of drug-resistant seizures refractory to valproic acid for 3 years prior to this admission suggests extensive parenchymal CNS involvement beyond the hypothalamic–pituitary axis alone. Third, the autonomic features of hypothermia and hypotension reflect additional dysfunction of hypothalamic thermoregulatory and cardiovascular centers. This triad of delayed neuroendocrine presentation, refractory epilepsy, and autonomic instability represents a particularly complex and instructive clinical phenotype not commonly described in the existing literature.

The patient’s presentation was notable for polyuria, polydipsia, hypernatremia, and hypothermia, all of which are hallmarks for diabetes insipidus and hypothalamic dysfunction [8]. Laboratory findings confirmed panhypopituitarism with deficiencies in cortisol, TSH, LH, testosterone, and elevated levels of prolactin which is likely due to pituitary stalk compression that leads to disruption in dopaminergic inhibition a phenomenon observed in both neoplastic and inflammatory sellar lesions [9]. Imaging revealed suprasellar and brainstem lesions consistent with prior reports of neurosarcoidosis involving hypothalamus and pituitary [10, 11]. The observed hypothermia and hypotension indicated additional hypothalamic dysfunction particularly in the regions preoptic and paraventricular nuclei responsible for thermoregulation and autonomic control [12]. Alongside the patient’s endocrine imbalance neurological symptoms were also present, most notably seizures that persisted even after valproic acid therapy. This resistance to treatment suggests extensive CNS involvement much like other documented neurosarcoidosis cases where controlling seizures demanded both immunosuppressants and antiepileptics [13]. Although cranial neuropathies are the most common neurological manifestation of neurosarcoidosis with occurrence in 50% of total cases, seizures are reported in less than 20% of the patients and often indicate parenchymal brain involvement or disease of the meninges [14].

Although the patient was maintained on prednisone and methotrexate at the time of admission, the presence of drug-resistant seizures and extensive CNS involvement raises the question of whether escalation of immunosuppressive therapy is warranted. Current evidence supports consideration of second-line agents such as azathioprine, mycophenolate mofetil, or rituximab in cases of refractory neurosarcoidosis unresponsive to first-line corticosteroids and steroid-sparing therapy. A formal reassessment of the immunosuppressive regimen was planned at outpatient follow-up in coordination with neurology and rheumatology. Given the potentially life-threatening consequences of unrecognized adrenal insufficiency, we recommend that all patients with suspected hypothalamic–pituitary neurosarcoidosis undergo early measurement of morning cortisol and, where indicated, a short Synacthen (cosyntropin) stimulation test, with prompt initiation of glucocorticoid replacement if insufficiency is confirmed.

Based on the 2018 Neurosarcoidosis Consortium Criteria (NCC), this case fulfills the criteria for probable neurosarcoidosis, given the prior biopsy-confirmed systemic sarcoidosis, compatible clinical and imaging findings, and exclusion of alternative diagnoses. Definite classification was not achievable in the absence of nervous system tissue biopsy. Although MRI is the preferred imaging modality for neurosarcoidosis, offering superior soft tissue resolution and the ability to characterize leptomeningeal enhancement, pituitary stalk thickening, and hypothalamic lesions, the prior MRI performed at an outside facility was unavailable for reproduction. We strongly recommend dedicated MRI of the brain and pituitary with gadolinium contrast as the first-line imaging investigation in all patients with suspected hypothalamic–pituitary neurosarcoidosis. Pituitary biopsy was not pursued for several reasons: neurosurgery consultation determined there was no safe surgical approach for pituitary biopsy, and the patient declined brain biopsy. However, alternative tissue diagnosis was successfully obtained via laryngeal biopsy performed by otolaryngology, which demonstrated granulomatous inflammation with chondronecrosis, providing strong supportive evidence for neurosarcoidosis without requiring nervous system tissue sampling. Additionally, the pre-existing biopsy-confirmed systemic sarcoidosis (skin biopsy, 2013) further supported the diagnosis. Serum ACE levels were elevated, providing additional evidence for active sarcoidosis. FDG-PET was considered but was not available at the time of this admission; it is acknowledged as a valuable adjunct in diagnostically uncertain cases to assess systemic disease activity and identify accessible biopsy sites.

A critical diagnostic challenge in neurosarcoidosis is possible mimicry of other conditions like pituitary adenoma, lymphoma, multiple sclerosis, or infectious granulomas [15, 16]. The patient’s prior sarcoidosis diagnosis and the exclusion of alternative etiologies as indicated by negative CSF analysis and no evidence of neoplasia supported neurosarcoidosis as the underlying cause. Additionally, the image findings observed in our case were in parallel with the 2018 neurosarcoidosis Consortium criteria which focus on the combination of clinical, laboratory, and radiographic findings for diagnosis when histopathological confirmation is not available [17]. Our patient was maintained on prednisone and methotrexate representing first-line therapy for systemic sarcoidosis [18]. The rapid response to desmopressin as indicated by elevated urine osmolality highlights the targeted diabetes insipidus management, while levothyroxine and glucocorticoid replacement addressed his central hypothyroidism and adrenal insufficiency. Despite the targeted intervention, the hormonal deficiencies in neurosarcoidosis are typically irreversible as observed in our patient and also reported in multiple cases in literature [19]. This persistence in hormonal dysfunction may be attributed to granulomatous inflammation that often leads to permanent disruption of pituitary tissue structure [20].

Neurosarcoidosis with hypothalamic–pituitary axis disturbance should be suspected when sarcoidosis patients develop polyuria, seizures, or endocrine dysfunction. Diabetes insipidus often appears first followed by anterior pituitary deficiencies that typically persist despite treatment. Refractory neurological symptoms may require advanced immunosuppression beyond steroids. Early hormone replacement prevents complications while multidisciplinary care optimizes outcomes. Additionally, given the potentially life-threatening consequences of unrecognized adrenal insufficiency, we recommend that all patients with suspected hypothalamic–pituitary neurosarcoidosis undergo early measurement of morning cortisol and, where indicated, a short Synacthen (cosyntropin) stimulation test, with prompt initiation of glucocorticoid replacement if insufficiency is confirmed.

## Conclusion

Neurosarcoidosis can cause permanent pituitary damage as shown by this case of hormone deficiencies and refractory seizures that developed years after initial diagnosis. Treatment requires prompt hormone replacement and customized immunosuppression. Clinicians must remain alert for these potentially delayed complications in all sarcoidosis patients including those previously stable.

### Learning points

- Neurosarcoidosis should be considered in sarcoidosis patients presenting with new-onset endocrine or neurological symptoms, especially polyuria, polydipsia, or seizures.
- Involvement of the hypothalamic–pituitary axis may result in panhypopituitarism and central diabetes insipidus, often requiring lifelong hormone replacement therapy.
- Diagnosis can be challenging due to its ability to mimic neoplastic, infectious, or demyelinating processes, and often relies on clinical, laboratory, and imaging findings in the absence of histopathology.
- Pituitary hormone deficiencies in neurosarcoidosis are frequently irreversible, emphasizing the importance of early detection and prompt endocrine evaluation.
- Multidisciplinary management involving endocrinology, neurology, and rheumatology is essential to optimize long-term outcomes in these complex cases.

## Data Availability

All data supporting the findings of this study are included in this article. No additional data are available.
